# Supplementary material for: Protein Complex Heterogeneity and Topology Revealed by Electron Capture Charge Reduction and Surface Induced Dissociation
Source: ACS Cent Sci. 2024 Jul 26;10(8):1537–47. doi: 10.1021/acscentsci.4c00461 (PMC11363329; doi:10.1021/acscentsci.4c00461)
Supplement: Supplementary file 2 — oc4c00461_si_002.pdf [file oc4c00461_si_002.pdf]

oc-2024-004615.R1

Name: Peer Review Information for "Protein complex heterogeneity and topology revealed by electron capture charge reduction and surface induced dissociation"

First Round of Reviewer Comments

Reviewer: 1

Comments to the Author

This manuscript combines gas-phase charge reduction with surface induced dissociation as a creative and powerful approach to characterize heterogeneous macromolecular complexes. It is a nice and comprehensive manuscript, and the technology holds a lot of potential. The applications provide a clear demonstration of the technology to important systems. It is a good fit for ACS Central Science, and I enthusiastically recommend it for publication after some revisions:

Much of the manuscript is written in very large, uninterrupted paragraphs. It would be nice to have more paragraph breaks everywhere. As written, it's harder to follow the flow of ideas in a dense paragraph.

Replicates and reproducibility are not discussed. The number of replicate measurements needs to be described in the methods sections. Also, it would be helpful to have some discussion (maybe with supporting data) on how reproducible the results are. For example, how much variation in charge reduction is there on different days?

It would be helpful to have a y-axis label on Figure S2.

I think it is important to briefly remind the readers that the quadrupole isolation only captures a small region of the total heterogeneous mixture and thus is only a small subset of the total species in the mixture. My concern is that readers may think there are only a few species in the data and that the quad isolation is magically helping to resolve them. Instead, my guess is that there could be a vast multitude of species, and the quad is just serving to pick out a handful of them at a time. Is it possible to either use a full coverage of the quad, similar to Sandoval et al., or an estimate from sparse coverage (as mentioned in the manuscript) to estimate the total number of features, keeping in mind that there may still be many combinations of nearly isobaric glycoforms that underly each

feature? For example, if you see 5 features from a quad selection window that is 1% of the peak, does that mean there are 500 features? Also, how reproducible are these features?

Reviewer: 2

Comments to the Author

This manuscript describes an approach for gas phase charge reduction based on electron capture. Electron capture charge reduction (ECCR) is demonstrated to improve the effective resolution of heterogeneous protein complexes, such as thyroglobulin and VFLIP spike glycoprotein. Its ability to mass measure extremely large and heterogeneous proteins is shown to compare well to mass photometry and Direct Mass Technology. When applied to surface induced dissociation (SID) of GroEL tetradecamers, ECCR was shown to yield product ion spectra that appeared more native-like in the population of multimers produced.

The manuscript will greatly interest readers engaged in native mass spectrometry. I recommend publication with only minor revisions.

Figure 2 caption: Panel J is a zoom… In the figure it is labeled as I.

Why is 4-mer is barely present in Fig. 3B, yet readily observed in Fig. 3D?

Page 2, lines 48-50. “The Smith group utilized corona discharge or alpha-particle sources to generate anions used for charge manipulation via proton abstraction of electrosprayed proteins.51,52” Because those authors manipulated charge with anions, rather than electrons, the sentence fits better with the previous paragraph. The “other” Smith (R. D. Smith) applied corona discharges for this same use ~1992 and should probably also be cited. Also, comprehensive examination of charge reduction by discharges was performed by Bornschein and Ruotolo (DOI: 10.1039/C5AN01242B).

Author's Response to Peer Review Comments:

Dear Editor:

We appreciate the reviewers' positive and helpful critiques of our manuscripts. We have attempted to address their comments in full, as described in the response to reviews. We have modified one of our main figures with new data and a Monte Carlo simulation (based on glycoproteomics) overlaid with the native MS data. We have made text clarifications throughout. The major changes are highlighted in the "for review only" highlighted version of the manuscript.

We appreciate your consideration of this manuscript for back-to-back publication with that of Sobott and coworkers (oc-2024-004627). Please let me know if you need any additional information.

Vicki Wysocki

We thank the reviewers for their careful reading and helpful comments on our manuscript. We have sought to address all comments within the paper as detailed here. Reviewer comments are given in black font, author responses are given in green, and copied text from the manuscript is given in blue.

Several minor wording changes were made throughout to improve the readability of the manuscript; these were not highlighted as tracked changes in the highlighted version of the document.

We also added one co-author, Regina Edgington, who initated the Monte Carlo simulations during a rotation in the Wysocki laboratory.

We appreciate your consideration of this manuscript for back-to-back publication with that of Sobott and coworkers (oc-2024-004627).

Formatting Needs:

ABSTRACT WORD COUNT: Please make sure the word count does not exceed 200 words. Reduced to 200 words

SI PARAGRAPH: If the manuscript is accompanied by any supporting information for publication, a brief description of the supplementary material is required in the manuscript. The appropriate format is: Supporting Information. Brief statement in non-sentence format listing the contents of the material supplied as Supporting Information. SI paragraph added

SYNOPSIS MISSING: The synopsis should be no more than 200 characters (including spaces) and should reasonably correlate with the TOC graphic. The synopsis is intended to explain the importance of the article to a broader readership across the sciences. Please place your synopsis in the manuscript file after the TOC graphic, and label it as “Synopsis.”

Synopsis added after the TOC graphic

Electron capture charge reduction and surface induced dissociation enable more native-like fragmentation and define heterogeneity in glycosylated protein complexes.

SI PAGE NUMBERS: Pages should be numbered S1, S2, etc

Page numbers added

Reviewer(s)' Comments to Author:

Reviewer: 1

Recommendation: Publish in ACS Central Science after minor revisions noted.

Comments:
This manuscript combines gas-phase charge reduction with surface induced dissociation as a creative and powerful approach to characterize heterogeneous macromolecular complexes. It is a nice and comprehensive manuscript, and the technology holds a lot of potential. The applications provide a clear demonstration of the technology to important systems. It is a good fit for ACS Central Science, and I enthusiastically recommend it for publication after some revisions:

Much of the manuscript is written in very large, uninterrupted paragraphs. It would be nice to have more paragraph breaks everywhere. As written, it's harder to follow the flow of ideas in a dense paragraph.

We have broken down the long paragraphs. We also made several minor wording changes throughout to improve overall readability.

Replicates and reproducibility are not discussed. The number of replicate measurements needs to be described in the methods sections. Also, it would be helpful to have some discussion (maybe with supporting data) on how reproducible the results are. For example, how much variation in charge reduction is there on different days?

We have added new supporting information, Figure S3 and Table S2, showing MS1 and ECCR of C-Reactive Protein acquired over 7 months. Figure S3 shows the amount of charge reduction is remarkably consistent when using the same voltage profiles over many months. Some of the variability in ECCR reflects variability in the MS1 charge state distribution .

The following sentence was added to the manuscript:

Figure S3 demonstrates long-term stability and high reproducibility of ECCR for CRP, where it was observed the standard deviation in the average charge state after ECCR (± 1.9) is slightly higher than the standard deviation in the average charge state before ECCR (± 0.9), over 7 months of data acquisition.

The following figure was added to the SI:

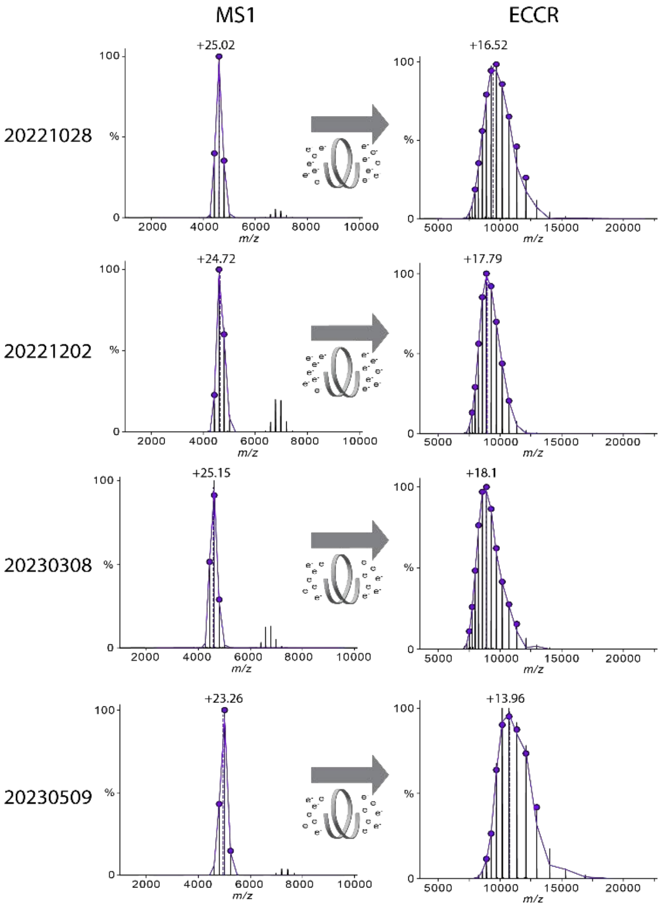

Figure S3. Long term stability and reproducibility of ECCR for C-Reactive Protein pentamer. The charge state distribution from m/z 4000 to 6000 was quadrupole mass selected and subjected to ECCR using the same voltage profile over 7 months. MS1 and ECCR charge state distributions are labeled with the weighted average charge. Replicates are shown over 7 months of use, and the average charge state before ECCR is +24.5 ± 0.9, while the average charge state after ECCR is +16.6 ± 1.9 (± refers to standard deviation). The voltages applied to the ExD-SID device are shown in Table S2. (Calendar dates of acquisition are shown on the left in year-month-day format.)

It would be helpful to have a y-axis label on Figure S2.

A y-axis label was added to Figure S1 and Figure S2

I think it is important to briefly remind the readers that the quadrupole isolation only captures a small region of the total heterogeneous mixture and thus is only a small subset of the total species in the mixture. My concern is that readers may think there are only a few species in the data and that the quad isolation is magically helping to resolve them. Instead, my guess is that there could be a vast multitude of species, and the quad is just serving to pick out a handful of them at a time. Is it possible to either use a full coverage of the quad, similar to Sandoval et al., or an estimate from sparse coverage (as mentioned in the manuscript) to estimate the total number of features, keeping in mind that there may still be many combinations of nearly isobaric glycoforms that underly each feature? For example, if you see 5 features from a quad selection window that is 1% of the peak, does that mean there are 500 features? Also, how reproducible are these features?

We agree with the reviewer's comments and have clarified in the manuscript that we are not capturing the full complexity of the sample when only three isolation windows are used. We have also simplified the figure below and added additional data, showing results from 10 narrow window isolations (inset of Fig 5F) compared with theoretical masses determined from a proteomics-based Monte Carlo simulation.
The new figure and modified discussion are shown below.

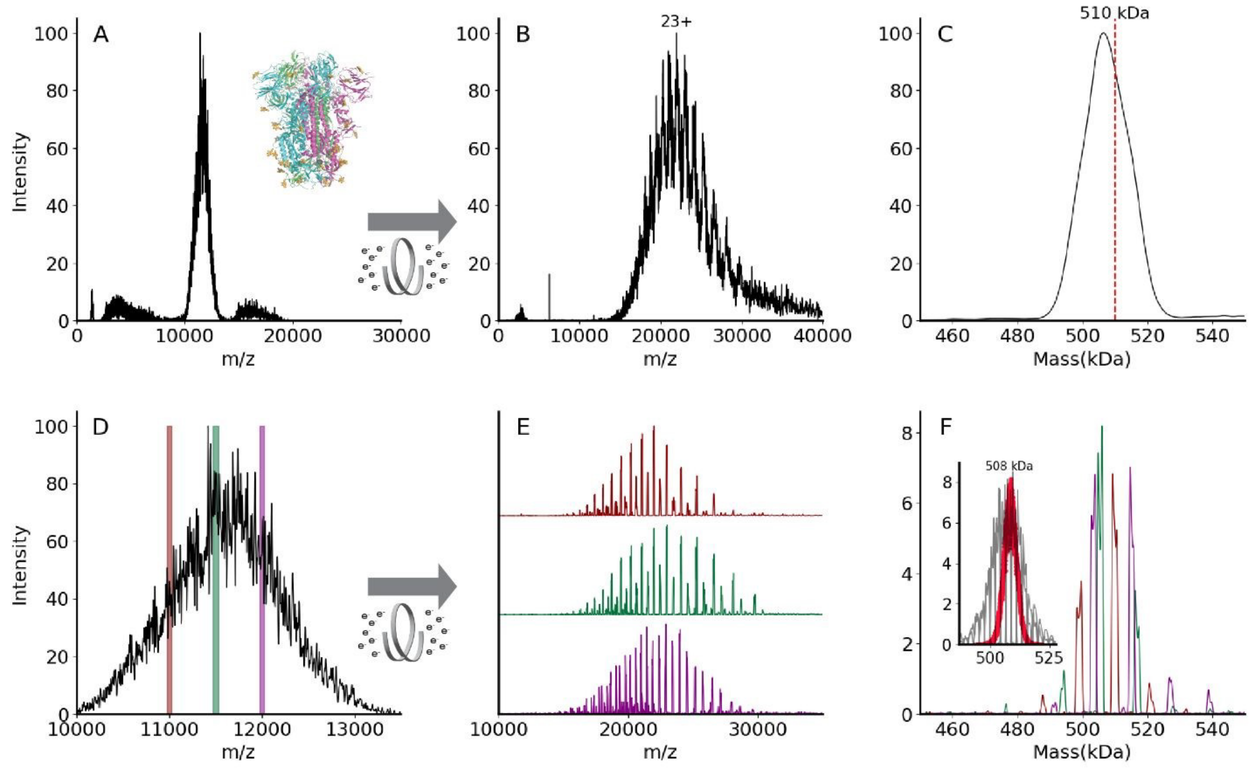

Figure 5. (A) An unresolved native mass spectrum of the heterogeneously glycosylated spike trimer VFLIP.[82] Inset shows the ribbon structure of SARS-CoV-2 spike trimer (PDB: 6X79). Three protomers are shown in pink, green, or blue, and N-glycans are shown in gold. (B) A charge state-resolved native mass spectrum of the spike trimer after electron capture charge reduction (ECCR, voltage 7V). (C) The average mass is 506 kDa deconvolved using UniDec parameters described in Methods.[89] The red dashed line **indicates** theoretical mass of 510 kDa. (D) Zoom in for spike trimer VFLIP from A with the positions of three narrow quadrupole selections shown. (E) Plots showing ECCR corresponding to the three narrow window isolations (10975-11025 (maroon); 11475-11525 (green); 11975-12025 (magenta), respectively. (F) Overlayed deconvolved mass spectra for data shown in panel E. The intensity was not normalized to better reflect the original intensity in panel D. The detected masses for first narrow window selection (maroon) are 499,645 Da, 509,355 Da, 520,689 Da; those for the second (green) are 494,331 Da, 504,835 Da, 506,070 Da, 516,276 Da, and 517,408 Da; those for the third selection (magenta) are 503,916 Da, 514,746, and 526,832 Da. Inset shows deconvolved masses from ECCR of 10 narrow isolation windows (50 m/z units wide, from 10,975-to 11,475 m/z) in gray with an overlay shown in red of the Monte Carlo simulations of theoretical masses based on the glycoproteomic data.

Three isolation windows do not capture the sample's full heterogeneity of the sample. To highlight the complexity and heterogeneity of this sample, we performed additional ECCR experiments on 10 adjacent narrow isolation windows (gray trace in the inset of Figure 5F) and compared the results to theoretical masses obtained by using a Monte Carlo simulation based on glycoproteomics data (red dots in the inset of Figure 5F). [90,91] The proteomics-based Monte Carlo simulation implies a broad mass distribution for spike protein,[90,91] with masses from 492 kDa to 522 kDa, with an average mass for spike protein of 508 kDa. However, the native MS ECCR results (gray) reflect a broader, more heterogeneous distribution. The broad distribution is like that reported by Jarrold, Clemmer, and Robinson by CDMS,[14] but our ECCR results and proteomics-based Monte Carlo simulation results center at around the same mass rather than at different masses and distinct glycoform masses are available from the *m/z* window slicing and

ECCR. There are several possible reasons that the width of the proteomics-based simulations and the native MS distribution might differ. 1) The difference may be caused, at least partially, by the different cell lines used for VFLIP samples (Chinese hamster ovary cell line) compared to those used for the glycoproteomics studies (Human embryonic kidney 293 cell line).[14,90,91] 2) The glycoproteomics data (digested proteins) may not provide information on biological glycan crosstalk, where the glycan type at one site may influence the glycosylation on another site. 3) The proteomics results may not capture all possible glycopeptides because of stochastic data collection, chromatography issues, and/or dynamic range issues. Based on our results, we suggest that this native MS method involving narrow-window *m/z* selection coupled to ECCR could be utilized as a quick screen for glycan complexity under different expression conditions for therapeutic proteins or various variants of concern in infectious diseases. This approach could be coupled with in-depth glycoproteomic studies (top-down d/or bottom-up) when the variable identities at each glycosylation site are required.

Additional Questions:
Quality of experimental data, technical rigor: High
Significance to chemistry researchers in this and related fields: Top 5%
Broad interest to other researchers: Top 5%
Novelty: Top 5%
Is this research study suitable for media coverage or a First Reactions (a News & Views piece in the journal)?: Yes

Reviewer: 2
Recommendation: Publish in ACS Central Science after minor revisions noted.

Comments:
This manuscript describes an approach for gas phase charge reduction based on electron capture. Electron capture charge reduction (ECCR) is demonstrated to improve the effective resolution of heterogeneous protein complexes, such as thyroglobulin and VFLIP spike glycoprotein. Its ability to mass measure extremely large and heterogeneous proteins is shown to compare well to mass photometry and Direct Mass Technology. When applied to surface induced dissociation (SID) of GroEL tetradecamers, ECCR was shown to yield product ion spectra that appeared more native-like in the population of multimers produced.

The manuscript will greatly interest readers engaged in native mass spectrometry. I recommend publication with only minor revisions.

Figure 2 caption: Panel J is a zoom… In the figure it is labeled as I.

We thank the reviewer for bringing this to our attention and have corrected the figure caption to reference panel I.

Why is 4-mer is barely present in Fig. 3B, yet readily observed in Fig. 3D?

As we and others have noted previously in the literature and here in the text, "normal" charge precursors do not produce native-like fragmentation patterns when subjected to SID. Reduction of precursor charge provides more native-like fragmentation patterns. The "missing" tetramer of CRP is either further fragmenting or not being produced because of the high charge on the precursor. Highly charged precursors sometime tend to expand/unfold in preference to native-like fragmentation and favor e.g., more monomer.

We have added material clarifying this to our discussion on solution phase charge reduction of CRP, as shown below.

SID of the entire charge state distribution of CRP at 40 V (energy range of 880 to 960 eV, determined by multiplying the SID voltage by the charge states observed above 5% relative intensity) produced primarily monomer and dimer, with lower levels of trimer and tetramer in agreement with previous studies (Figure 3B).[69,79] We selected the full charge state distribution knowing that we won't be able to select an individual charge state after ECCR, because the ExD-SID device is after the quadrupole mass filter.

For a cyclic complex like CRP, we expect all oligomeric states between monomer and tetramer at low SID energies, with relatively high abundance due to the equal interfaces between all subunits. Solution charge reduction of CRP (160 mM ammonium acetate and 40 mM triethylammonium acetate) yielded a charge state distribution with a weighted average charge of 18+ (Figure 3C). SID of this charge state distribution at 60 V (energy range of 1020-1140 eV) produced [monomer and tetramer] and [dimer and trimer] at high intensity (Figure 3D) consistent with the native cyclic structure and with previous SID studies of CRP.59,68,80 As noted above, charge-reduced precursors are often chosen for native SID studies as the fragmentation has been observed to be more native-like producing more of the higher-order oligomers due to decreased secondary fragmentation and/or unfolding.68,78

Page 2, lines 48-50. "The Smith group utilized corona discharge or alpha-particle sources to generate anions used for charge manipulation via proton abstraction of electrosprayed proteins.51,52" Because those authors manipulated charge with anions, rather than electrons, the sentence fits better with the previous paragraph. The "other" Smith (R. D. Smith) applied corona discharges for this same use ~1992 and should probably also be cited. Also, comprehensive examination of charge reduction by discharges was performed by Bornschein and Ruotolo (DOI: 10.1039/C5AN01242B).

We have added reference to the Ruotolo and R.D. Smith papers and moved reference to the Smith group studies. The relevant sections of the manuscript are copied below.

Commercially, PTR and the related proton transfer charge reduction (PTCR) has been offered on various instrument platforms.[28–33] Ogorzlek Loo et al. demonstrated charge reducing capabilities of corona discharge generated anions during the electrospray process.[34] The Smith group utilized corona discharge or alpha-particle sources to generate anions used for charge manipulation via proton abstraction of electrosprayed proteins.[35,36] Similarly, Bush and coworkers have used a glow-discharge source to generate anions for PTR with *m/z*-selected ions of native proteins and complexes to enable charge assignment and mass determination.[37] Additionally, Bornschein and Ruotolo studied how charge reduction via corona discharge generated anions affected the collisional ejection of subunits from protein complexes. Sandoval and coworkers have recently demonstrated the utility of PTCR and gas-phase fractionation for the analysis of intact glycoproteins.[38]

Additional Questions:
Quality of experimental data, technical rigor: Top 5%

Significance to chemistry researchers in this and related fields: Top 5%
Broad interest to other researchers: High
Novelty: High
Is this research study suitable for media coverage or a First Reactions (a News & Views piece in the journal)?: No
